# Synchronous robotic right hemicolectomy and subtotal gastrectomy

**DOI:** 10.1007/s13304-020-00866-8

**Published:** 2020-08-27

**Authors:** Fabio Carbone, Ugo Pace, Vittorio Albino, Maddalena Leongito, Paolo Delrio

**Affiliations:** 1 Department of Advanced Biomedical Sciences, University of Naples “Federico II”, Corso Umberto I 40, 80138 Naples, Italy; 2 Colorectal Surgical Abdominal Oncology Department, Istituto Nazionale per lo Studio e la Cura dei Tumori “Fondazione Giovanni Pascale” IRCCS, Via Mariano Semmola 52, 80131 Naples, Italy; 3 Gastro-pancreatic Surgery Department, Istituto Nazionale per lo Studio e la Cura dei Tumori “Fondazione Giovanni Pascale” IRCCS, Via Mariano Semmola 52, 80131 Naples, Italy

**Keywords:** Robotic surgery, Right hemicolectomy, Gastrectomy, Colon, Stomach, Cancer, Technique

## Abstract

About 4% of patients with stomach cancer diagnosis have synchronous colorectal cancer and some of these patients may require a synchronous surgical resection. So far, only few minimally invasive series of synchronous resections have been described. We investigated the feasibility and safety of the synchronous robotic resection of the right colon and stomach malignancies, trying to identify a standardised and reproducible technique. It is essential to carefully plan the operation and the trocars positioning to minimise the number of robotic dockings and be able to operate comfortably. Herein, we describe our approach, which is safe and effective in terms of minimal invasiveness and oncological radicality. Robotic surgery could be used with even more advantage in complex multi-organ resections, providing the surgeon with a better vision, a more accurate dissection and longer instruments, to offer the patient all the benefits of a minimal invasive surgery.

## Introduction

About 4% of patients with stomach cancer diagnosis have synchronous colorectal cancer and some of these patients may require a synchronous surgical resection. [1] The feasibility and safety of robotic right hemicolectomy and partial gastrectomy as minimally invasive techniques have already been proven since 2002 [2] and 2008 [3], respectively. So far, only a few laparoscopic [4–6] and one robotic [7] case series of synchronous resections have been described. Especially in multi-organ resections, robotic surgery may give a significant gain in terms of postoperative recovery and ease of procedure for the surgeon. Furthermore, during COVID-19 (Coronavirus disease-19) pandemic era, the use of the robot for complex oncologic surgery could provide a safety advantage for health professionals and patients. Although there are several techniques and different approaches for both robotic right hemicolectomy and partial gastrectomy, we investigated the feasibility and safety of the robotic synchronous resection of the right colon and stomach malignancies, trying to identify a standardised and reproducible technique.

## Case presentation, set-up and access

We present the case of a 63-year-old man with BMI of 30.5, who presented with a right colic flexure adenocarcinoma and an early tumour in the great curvature of the gastric antrum diagnosed at the colonoscopy and esophagogastroduodenoscopy (EGD), respectively. After complete staging with a CT (Computed Tomography) scan, the case was discussed within the multidisciplinary team and a synchronous colon and gastric cancer surgery was planned. The right colon cancer was marked endoscopically and staged as ctT_3_N_0_ according to AJCC (American Joint Committee on Cancer) 8th edition. The patient was assessed as ASA (American Society of Anesthesiologists) score 2 at the anaesthetic evaluation. Since the patient came to our attention during the COVID-19 pandemic outbreak, he also had a swab test for COVID-19 performed and resulted negative. Due to the complexity of this cancer surgery and the COVID-19 pandemic situation, we opted to perform a synchronous robotic right hemicolectomy and partial gastrectomy with D2 lymphadenectomy using the Intuitive Da Vinci Xi^®^ system. [8].

The Da Vinci Xi^®^ has great versatility, but sometimes performing surgical procedures in two different abdominal compartments can be challenging. The operative set-up and positioning of the trocars is crucial to optimise the number of dockings necessary to complete both resections of the right colon and distal stomach with adequate lymphadenectomies. [9] Our approach pointed to find a single positioning of the trocars, suitable for both the sub-mesocolic and the supra-mesocolic phases, with only two dockings needed.

**Fig. 1:**
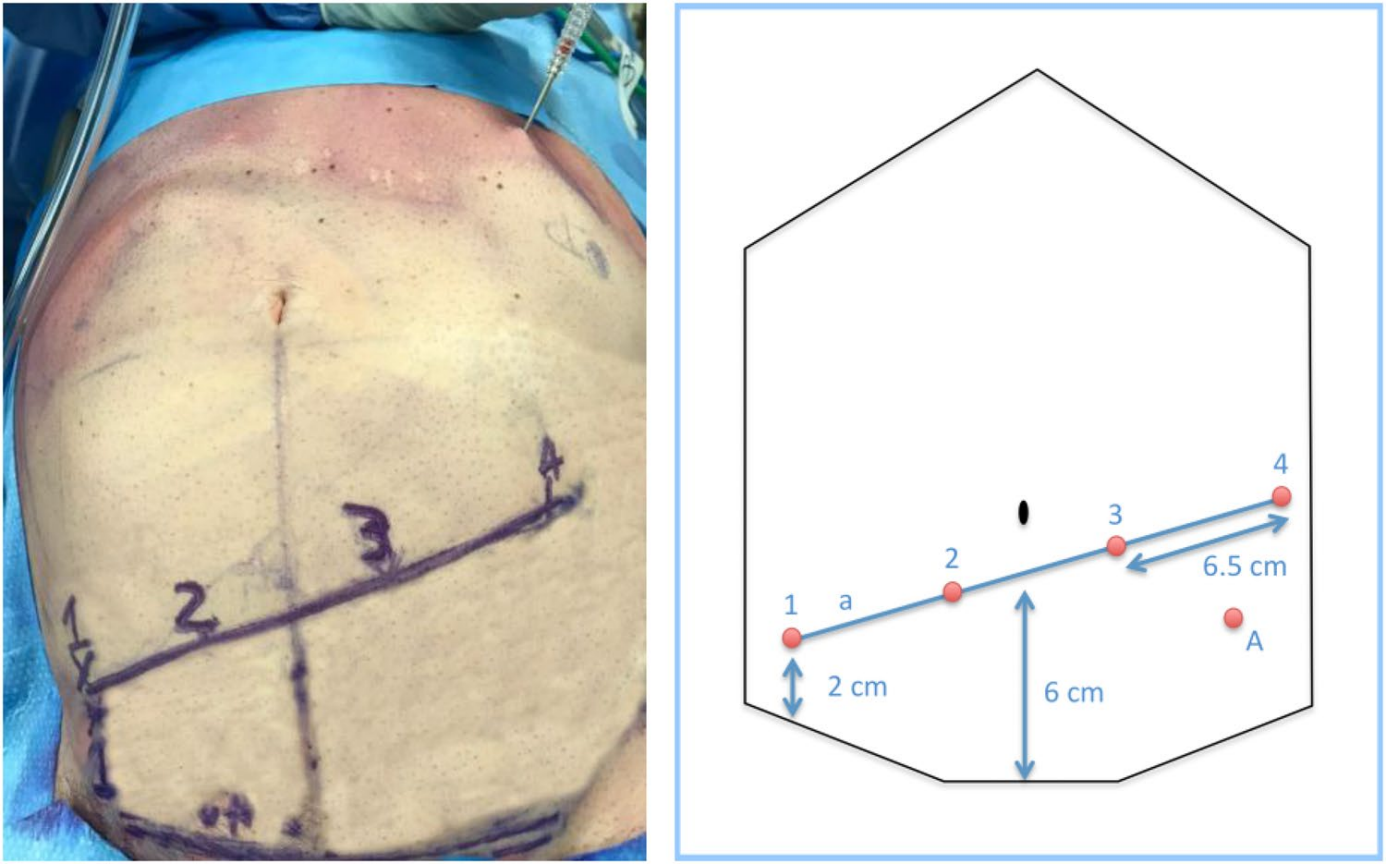
On the left, the pneumoperitoneum is induced using the Veress needle and the port placement is planned; on the right, schematic picture of trocar positioning. 1–4: robotic arms numbers; **a** the line on which the trocars are placed; A: assistant trocar

After inducing the pneumoperitoneum with the Veress needle at a pressure of 14 mmHg, four 8-mm trocars and one assistant 12-mm trocar with AirSeal^®^ system were used. The four 8-mm trocars were positioned on a line “a” passing through two points: the first point was 2 cm above the upper right anterior iliac spine, the second point was on the midline, 6 cm above the pubic line. Trocar number 1 was positioned exactly in the first point described above and the other three were positioned on the line “a” at a distance of 6.5 cm from each other. We believe that further distance between trocars could be considered depending on the size and the shape of the abdomen. The 12-mm assistant trocar (A) was positioned below the line “a”, equidistant from the trocars 3 and 4 on the left flank. All measurements were calculated after the pneumoperitoneum induction (Fig. 1).

## Right Hemicolectomy

The operating table was set in Trendelenburg position with an angle of 25–30°, with a left lateral inclination of 20°. The Da Vinci Xi^®^ patient cart was docked from the right side of the patient, the camera was inserted on the arm number 3, the right colic flexure was used as targeting point and the other instruments were inserted under vision: the Cadiere forceps on the arm 1, the fenestrated bipolar forceps on 2 and the monopolar curved scissors on 4 (Fig. 1).

**Fig. 2:**
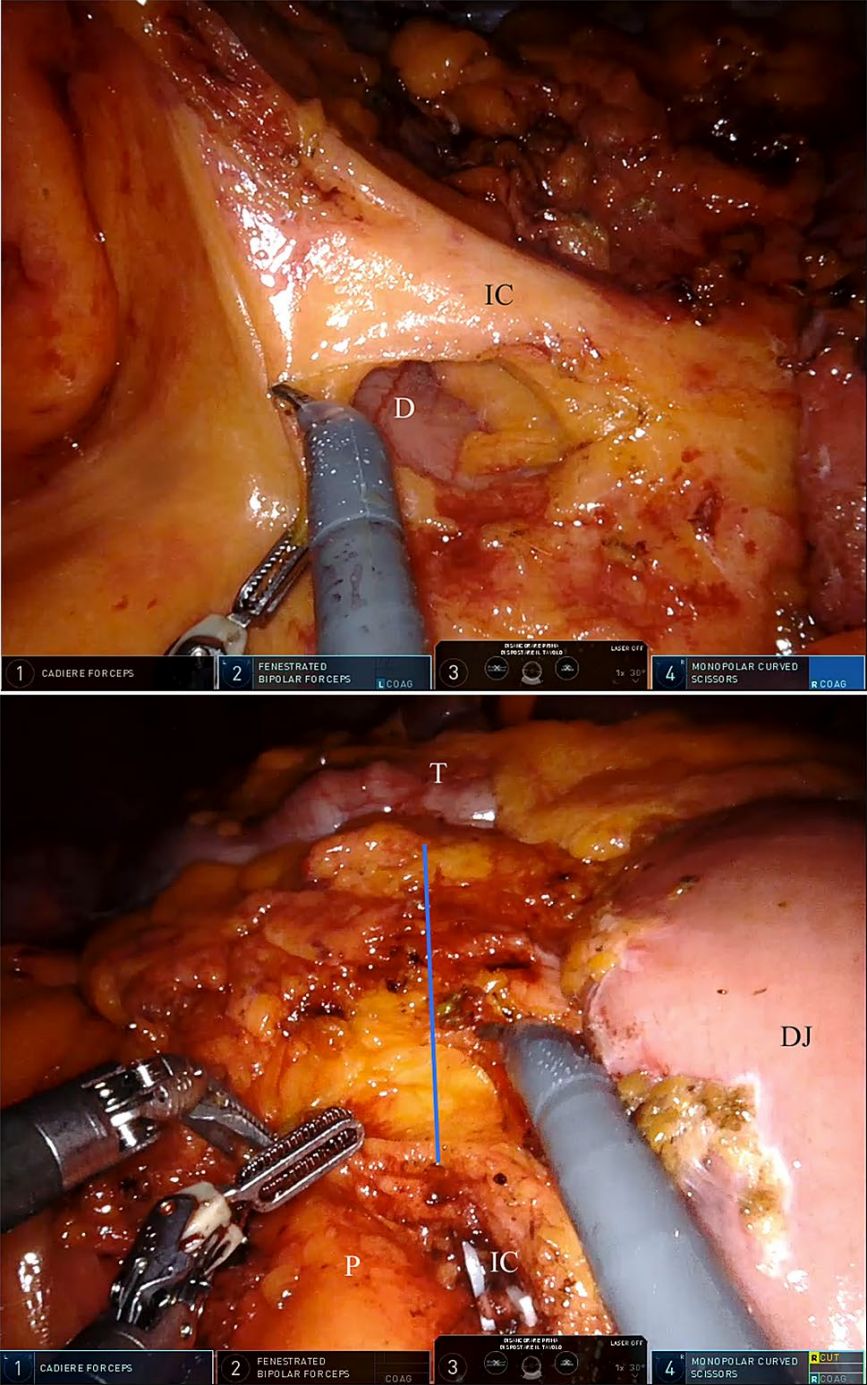
Above, identification of the ileocolic vessels (IC) and opening of the mesocolon on the left side of the IC allow the access to the duodenum (D); below, the transverse mesocolon is opened along the blue line, starting above the IC until the transverse colon (T) serosa. *P* pancreas, *DJ* duodenum–jejunal flexure

The ileocolic vessels were identified, dissected and ligated. The right colon was mobilised with a medial-to-lateral dissection from the Gerota’s fascia and the right parietocolic gutter. The transverse mesocolon was sectioned above the ileocolic vessels up to the transverse colon serosa and the right branch of the middle colic artery was identified, ligated and sectioned (Fig. 2). The transverse colon was sectioned with the SureForm^®^ 60 mm stapler (blue charge) distally to the tumour. The terminal ileum was also divided with the same instrument. Full mobilisation of the right colon was then completed by dissecting the right part of the gastrocolic ligament and the transverse mesocolon. The ileo-colic anastomosis and the extraction of the specimen were performed during the open phase. The Xi^®^ cart was undocked and set up for the supra-mesocolic surgery.

### Subtotal Gastrectomy

**Fig. 3:**
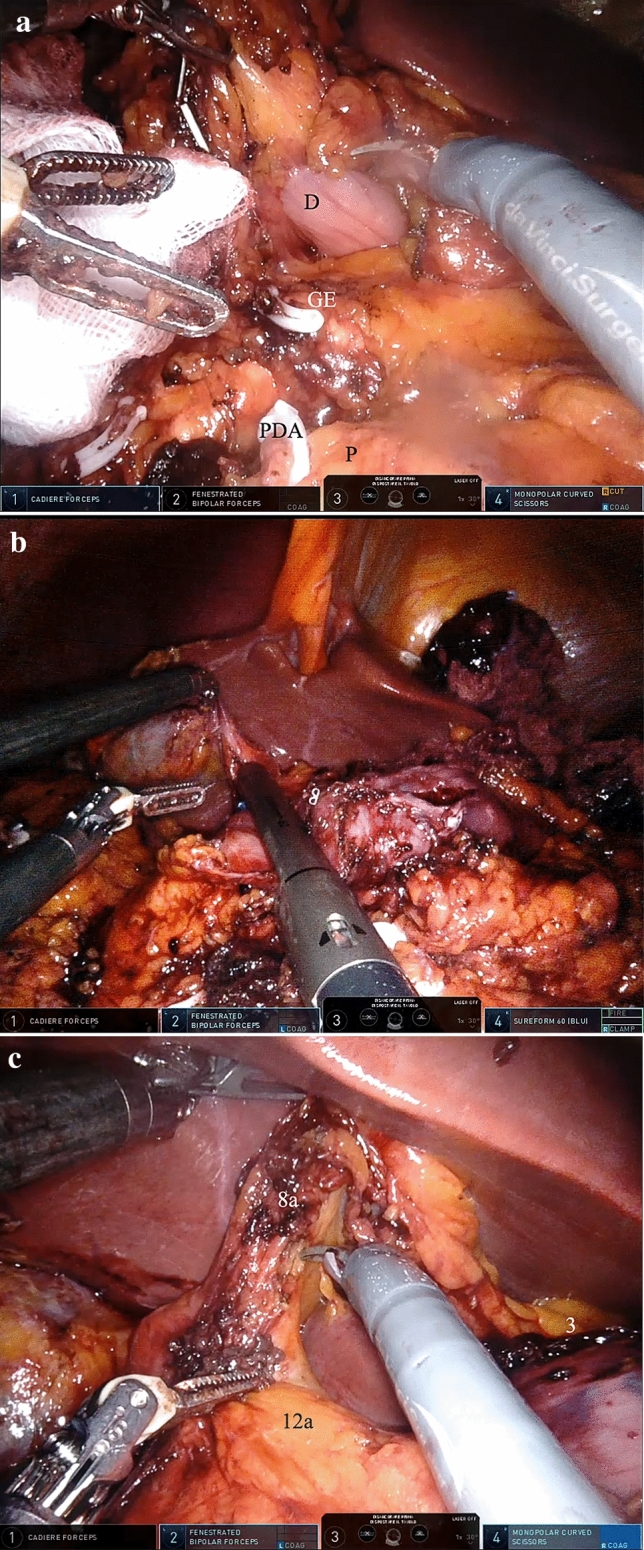
a Dissection and section of the pancreatico-duodenal artery (PDA) and gastroepiploic artery and vein (GE); **b** the duodenum (D) is tunnelled and sectioned using the robotic stapler Sureform® 60 mm; **c** lymphadenectomy of the stations 8a, 12a and 3 is performed

The operating table was set in reverse-Trendelenburg position with an angle of 25° and a slight left lateral inclination. The Xi cart was docked, the antrum of the stomach was used as targeting point and the instruments were inserted with the same pattern as described above. We proceeded mobilising the duodenum and the posterior side of the stomach using the gastrocolic window opened from the previous operating time. The pancreaticoduodenal and right gastroepiploic vessels were clipped and sectioned and the dissection of the 6th lymph node station was carried out (Fig. 3a). The hepatogastric ligament was opened and the full mobilisation of the first portion of the duodenum led to a safe stapling of the latter with the robotic Sureform^®^ 60 mm (blue charge), safely far from the bile duct (Fig. 3b). The lymphadenectomy continued on the anterior aspect of the hepatic artery, with the dissection of the lymph node stations 8a and 12a (Fig. 3c). The gastrocolic ligament opening was completed on the left side, leading to a full mobilisation of the distal part of the stomach along with the harvesting of the lymph node stations 4sb and 4d. Ligation and section of the left gastric artery, together with the lymphadenectomy of the 7th and 9th stations, were performed.

After desufflation and de-docking of the patient cart, an 8 cm median peri-umbilical incision was made, useful for both the extraction of the specimens and the anastomoses performance. A wall protector was placed and a gastro-jejunal trans-mesocolic mechanical anastomosis was performed 5 cm upstream to the tumour, using the 29-mm circular stapler. The gastric specimen was extracted. Afterwards, a jejunal–jejunal latero-lateral mechanical anastomosis was fashioned according with the Roux-en-Y technique. The colic specimen was extracted, the ileo-colic anastomosis was performed with a three line linear stapler and the abdominal wall was closed. The nasogastric tube was placed in the efferent jejunal bowel, downstream the gastro-jejunal anastomosis. One single drain was placed close to the duodenal stump.

## Results

The overall operating time was 380 min: the access and the two dockings time lasted 40 min, the robotic phase 290 min, the open phase 50 min. The blood loss was minimal. The patient was mobilised 6 h after the surgery. On the first postoperative day, the nasogastric tube was removed and the patient started drinking clear fluids. On postoperative day 3, the patient started a liquid diet and a solid diet on postoperative day 4. The drain was then removed and the patient was discharged on the postoperative day 5. No complications were recorded.

The colonic histopathology report showed a moderately differentiated adenocarcinoma, staged pT_3_N_0_V_0_R_0_G_2_ (AJCC 8th edition); 25 lymph nodes isolated with no evidence of metastasis.

The gastric histopathology report showed a high-grade dysplasia/intraepithelial adenocarcinoma, staged pT_is_N_0_R_0_G_1_ (AJCC 8th edition); 21 lymph nodes isolated with no evidence of metastasis.

The patient was re-evaluated one and three months after surgery and showed a full recovery with no signs of recurrence at the CT scan and tumour markers. No adjuvant chemotherapy was administered to the patient.

## Considerations

We have found several advantages in carrying out this combined operation with the robotic approach. First of all, the minimally invasive robotic approach allowed a fast postoperative recovery with little pain for the patient. The Da Vinci Xi^®^ allowed a minimally invasive approach to a surgery that otherwise would have required a very extensive abdominal incision, due to the tumours location in two different compartments of the abdomen. Although the laparoscopic approach for this synchronous operation has already been described in very small series of cases [4–6], we believe that the robotic approach has also the advantage of providing the surgeon with a better vision, a more stable operating field, a more accurate dissection and longer instruments that allow to easily reach the most distant points of the surgical dissection. Indeed the operation planning time and the correct positioning of the trocars are essential for a successful procedure.

Another advantage of the robotic approach is the use of a closed system for the discharge of the pneumoperitoneum gases, such as the AirSeal^®^ device, which provides a safety solution for healthcare professionals in the COVID-19 pandemic era [8].

The disadvantages of this technique could be the longer operating time for surgeons with little experience in robotic surgery [10] and the double docking necessary in the described technique, although new evidence would suggest the possibility of moving the operating table even with the Xi cart docked to the patient. [11].

Preoperative endoscopic marking of both lesions is obviously crucial to overcome the lack of tactile sensation with the robotic technique.

## Conclusions

Synchronous tumours of the right colon and distal stomach are relatively rare. Our approach for synchronous robotic right hemicolectomy and partial gastrectomy for colon and distal stomach cancer is safe and effective in terms of minimal invasive surgery and oncological radicality. It is essential to carefully plan the operation and the trocars positioning to minimise the number of robotic dockings and be able to comfortably perform the operation. Robotic surgery could be used with even more advantage in complex multi-organ resections, providing a better vision, a more accurate dissection and longer instruments to allow a minimally invasive approach. Further studies will clarify whether and how much this advantage is superior to the individual resections with other approaches.

## Data Availability

Not applicable.
